# Animal Odor

**Published:** 1856-06

**Authors:** Charles Van Allen


### SUGGESTIONS UPON ANIMAL ODOR.

BY CHARLES VAN ALLEN, M. D.

Each genus, species, and perhaps individual, of the whole world of animal life, is probably distinguished by its peculiar odor. We will not discuss with the metaphysician the question, whether odor exists independently of the sense of smell; we take it for granted that it is a property of certain forms of matter, independent of this sense. This fact, so apparent in animals that come within the ordinary scope of observation, justifies the conclusion by analogy, that the whole animal kingdom is similarly endowed. Some of the more prominent examples will naturally suggest themselves to the student of Natural History. The musk odor, with its numberless varieties, belonging to the musk deer (moschus moschiferous) to several of the ape kind; and to the different varieties of the musk rat; the peculiar smell of the civet, that of the castor (castor fiber) and the strong offensive odor of the pole cat and of our native skunk; and among insects, the delicate musk perfume of the *cerambix moschatus*, the *apis fragrans* and the *tipula moschifera* and the odor of the *cerambix saveolens*, nearly allied to, though more delicate than, the rich perfume of the otto of roses,—offer ready illustrations of the extent and variety of animal odor. In man, as in other animals, there exists a peculiar distinctive odor, not impressing itself in a state of health with much intensity upon our duller sensation, but always clearly perceptible to the more acute sensibility of some of the lower animals. It is contended with some degree of plausibility, that each race of mankind has its distinguishing odor. "The Peruvians," says Humboldt, "who in the middle of the night, distinguish the different races by their quick sense of smell, have formed three words to express the odor of the European, the American Indian, and the Negro." The following observation is quoted by Blumenbach from *Thibault de Chambollon*, in reference to the Caribleans. "They have all a strong and disagreeable smell. I cannot," says Chambollon, "give the remotest idea, by description, of its peculiarity. Whenever a similar odor is observed, it is called at the Antilles, the Caribbean smell, which proves the difficulty of defining it." It is generally conceded, and most of us have daily opportunity for obtaining olfactory evidence of the fact, that the negro emits a peculiar odor, distinguishing him from the white race. Dr. Carpenter denies this. He allows that the negro, in common with the Hindoo, secretes a more abundant perspiration than the white, but asserts emphatically that it is not more odoriferous. Dr. Prichard, on the other hand, though an earnest advocate for the unity of origin of the whole human family, remarks in speaking of the perspiration of the dark-colored races, that it has a peculiar odor, which is well known in negroes and the Caribbee Indians. On the score of smell, no obstacle exists to the most intimate relations between the white man and the negro, which will not easily yield to a due application of soap and water.

That each individual as well as race, of the human family, is endowed with a peculiar odor, seems evident, from the case with which the dog scents his master. The interesting history of Julia Brace, an inmate of the Hartford Asylum, affords further evidence of the fact. This unfortunate girl, born blind, deaf and dumb, having no other mode of communication with the external world than by the senses of smell, taste, and touch, by the increased power of which nature strove to compensate her for her severe affliction, was enabled to distinguish persons, by the exercise of the first sense alone. The eloquent author of the "Religio Medici," and the "Urn Burial," has some observations pertinent to this subject. "We acknowledge," he says, "that certain odors attend on animals, no less than certain colors; that pleasant smells are not confined to vegetables, but found in divers animals, and some more richly than in plants; and though the problem of Aristotle inquires, why no animal smells sweet beside the *pard*, yet later discoveries ad divers sorts of monkeys, the civet cat and jazelon, from which our musk proceedeth. We confess that beside the smell of the species, there may be individual odors, and every man may have a proper and peculiar savor, which, although not perceptible unto man, who hath this sense but weak, is yet sensible unto dogs, who thereby can single out their masters in the dark. We do not deny that particular men have sent forth a pleasant savor, as Theophrastus and Plutarch report of Alexander the Great, and Tzetzes and Cardon do justify of themselves." In that charming specimen of self-portraiture, the autobiography of Lord Herbert of Cherbury, the brave knight, the courteous gentleman, and the learned scholar, presenting in his character the rare union of a complete knowledge of the world and the deepest wisdom of the closet, we read, "It is well known to those who wait in my chamber, that the shirts, waistcoats, and other garments I wear next my body, are sweet beyond what easily can be believed or hath been observed in any else; which sweetness also was found to be in my breath above others, before I used to take tobacco, which, toward my latter days, I was forced to take against certain rheums and catarrhs that troubled me, which yet did not taint my breath, for any length of time." In a quaint old work entitled "The life of the learned and pious Dr. Henry More, late fellow of Christ College in Cambridge, by Richard Ward, A. M., the author remarks, "I was mentioning somewhat but just now of his body, and this reminds me of some things that were peculiar in that also as well as in his mind; he has told us occasionally, in a discourse concerning the famous Greatrakes and what was extraordinary in that person," "that not only his own urine had naturally the flavor of violets in it, but that his breast and body, especially when very young, would of themselves in like manner send forth flowery and aromatic odors from them, and such as he daily almost was sensible of, when he came to put off his clothes and go to bed ; and even afterwards, when he was older, about the end of winter or beginning of spring, he did frequently perceive certain sweet and herbaceous smells about him, when yet there was no such external objects near, from whence they could proceed."* These reports of sweet smells must be taken "cum grano salis," to give them the savor of truth. They probably take their origin in an exaggerated egotism, which envelopes in its incense everything that pertains to self.

In considering the physiology of animal odor, and endeavoring to trace out its cause, considerable difficulty is encountered from the unsubstantial nature of the object of investigation, from the impossibility of discriminating with exactness its varieties, and from the various degrees of sensibility of different observers. Attempts have been made to classify systematically the various odors. The most plausible one divides them into *Acidulous*, *Spirituous*, *Camphorous*, *Fragrant*, *Somniferous*, *Fetid* and *Alkaline ;* but these terms do not admit of a strictly philoshphical application. All odors are composite, and all individuals are possessed more or less of an idiosyncracy of sense. These facts present themselves as insuperable obstacles to an exact definition. The physician, in this as in every other department of the science of medicine, must not confide too trustingly in fixed rules, but endeavor to sustain his uncertain steps by individual experience and observation.

Various animals (of which mention has already been made) more prominently distinguished by their odor, such as the musk deer, the civet, and the castor, have peculiar organs, generally situated in the neighborhood of the genitals, devoted to the particular function of generating odor. In others, as the pole cat and the skunk, the stench is due to the ordinary secretions. The odor of these various animals is not of the same intensity at all times and seasons. With some it is voluntary, and thrown out as a protection against attack ; and with others it is generated independently of the will, in the rutting season, as a source of attraction between the sexes. Animal odor in its natural condition, of whatever kind, is unquestionably a source of pleas-

---

* In a late work, called the Table Talk of Samuel Rogers, we find the following: "Sir Henry Englefield had a fancy (which some greater men have had) that there was about his person a natural odor of roses and violets. Lady Greenville hearing of this, and loving a joke, exclaimed one day when Sir Henry was present, 'Bless me, what a smell of violets!' 'Yes,' said he, with great simplicity, 'it comes from me.'"

ure to its possessor, and to those of the same order, and of repugnance to most others, which contributes towards preserving the integrity and individuality of the races, thus administering to a wise purpose in the economy of nature.

The principal sources of odor in man, are the breath and the different secretions. The air expired gives out the odor, in diminished intensity, of the ordinary articles of diet consumed. The perspiration has naturally an acid odor, differing in degrees in various parts of the body, being more sensible in the groin, in the neighborhood of the genitals, and the feet. The other secretions and excretions have peculiar smells of their own, each of which contributes towards producing in man his distinctive odor. This is affected in a remarkable manner, and very variously, by habits of life, the atmosphere, diet, and more especially disease. Persons engaged in certain employments, whatever may be their attention to personal cleanliness, are observed to become so impregnated with the peculiar odor of the objects by which they are constantly surrounded, as to give it out long after a change of occupation. Chomel states that he had a hostler under his charge at the hospital, who during the course of a billious fever, exhaled a strong smell of the stable, although all his clothes had been removed, and he had been repeatedly washed. All who approached him felt assured that the odor proceeded directly from the patient himself.

The effect of diet and of various articles used as remedies, in altering the animal odor, is a familiar fact. Those in the habit of eating garlic, and onions, habitually smell of the peculiar odor of these plants ; and the Greenlander, whose constant vegetable food is of the *pea* kind, is reported by travelers to emit a leguminous smell; the noisome stench which infects the urine after eating asparagus cannot have escaped the dullest sense of smell ; and the violet odor from the administration of the oil of turpentine, the peculiar flavor given to the breath and urine and the perspiration by copaiba, sulphur, and various other remedies, are the results of everyday observation. It is doubtless the vapor of the essential oil belonging to the articles taken into the system, which is thus exhaled, and which, though not sensible to chemical tests, becomes evident the sense of smell. The varieties of odor among individual races are attributable probably to their various modes of life.

The effect of disease in increasing and varying the animal odor of the human body, more especially recommends itself to the regard of the medical observer, from its direct bearing upon diagnosis. It is believed that much useful information would be the result of directing inquiry and observation upon this subject. Its investigation is urged upon the physician as promising to result in a fair return of profit, available for practicable purposes. Certain diseased conditions of the system affect in a remarkable manner the odor of the breath, and thus afford a valuable symptom of their existence. In some febrile diseases the breath acquires a sweetish, and in various affections of the stomach a strong pungent, acid odor. This acidity is occasionally of such intensity, as to impregnate everything which surrounds the patient, his clothes, bedding, and the whole furniture of the room; and is indicative of a severe form of gastric disease, which most frequently results in death. In gangrene of the lungs, the breath emits an odor of putrified flesh, a most unvarying and important distinctive symptom of this disease. In several forms of dyspepsia, in billious fever, in scurvy, and in the latter stage of consumption and typhus fever, it assumes a fetid character. Mercurial salivation, and various affections of the mouth and throat are easily discernible by the peculiar and offensive odors they give to the breath. A fit of drunkenness is invariably detected by the spirituous odor, exhaled from the lungs, when otherwise there might be danger of confounding it with severe disease. The perspiration in a variety of different affections undergoes sensible changes in odor, worthy of the physician's regard. In prolonged constipation, a very marked odor of sulphureted hydrogen is obsevered, especially in females, who are more apt to neglect the state of their bowels, and whose false modesty disposes them to conceal their condition from their physician. This odor will be found a useful, and often the only attainable indication in such cases.

In various diseases of the skin, a peculiar smell of the transpiration is an unvarying symptom. In all syphilitic eruptions, the odor is marked and peculiar. In small-pox it is equally distinctive and prominent, and is by many compared to the smell of mouldiness; in porrigo favosa also, it resembles the stench of cat's urine, and in miliary fevers, by some it is likened to the smell of lime, by others, to that of decayed straw. A peculiar fetid smell of the perspiration of the feet is frequently observed, and presents a troublesome and obstinate disorder. The writers upon the sweating sickness, the terrible plague which devastated Europe in the 15th and 16th centuries, have exhausted their powers of language, in endeavoring to describe the rankness of the odor of the perspiration, which was the prominent symptom in that disease. They speak of the horrible stench of the sick, the *odor teterimus*, of the afflicted—being surrounded by a thick stinking mist—of their lying, as it were, in a stinking swamp of sweat, and overwhelmed with disgust of themselves, in consequence of their loathsome and ill-odored condition. In rheumatic diseases, most of the secretions and excretions are perceptibly changed in odor, and more especially the sweat, which assumes a nauseous acid smell. Fevers of a low typhoid character are easily indicated by an odor like that of mice; and in the latter stages of typhus, the smell is decidedly cadaverous. The insane are observed to emit a peculiar odor from the skin. The urine also acquires various odors in different diseases. In Bright's disease it sometimes exhales the smell of boiled beef; and in diseases of the bladder and some typhoid affections, that of shell fish; in acute inflammatory disease, and in various disorders of the kidneys, an ammoniacal odor. The smell peculiar to the fæces varies frequently in disease. It is of a cadaverous nature in typhoid fever and chronic diarrhœa, and resembles that of macerated flesh in certain malignant dysenteries. In many of the diseases of the digestive organs in children, its condition supplies important indications. The stools are offensive in the early stages of cholera infantum, and again inodorous in its more advanced periods, and in the severe forms of dysentery. The odor of what is thrown up in vomiting aids in forming a just notion of the nature of the disease in which it exists. The sense of smell is necessarily applied to for a right appreciation of the condition of certain ulcers, putrescent wounds, gangrene, and purulent deposits. The odor is the most reliable means for the detection of poisons by prussic acid, and aids in discovering the presence of the metallic, narcotic, and other poisons. The septic condition generally reveals itself by a smell indicative of the process of decomposition. It is to this state that may be attributed the various changes in the animal odor, produced by disease. Whether this disorganization originates in the solids or fluids, or, as is more probable, occasionally in both, it becomes no one in the present state of pathological science dogmatically to decide. That the composition of the humors of the body, is at least secondarily affected, there need be no hesitation in asserting.

It is no longer heresy to speak of the corruption of the fluids, the decomposition, or, in other words, the new chemical combinations set up in them, uncontrolled by the vis vitæ. To what else can be attributed the loathsome putrid sweats in the sweating sickness, the cadaverous odor in the last stages of typhus, and the putrescent exhalations in scurvy and other analagous affections? Much progress in pathology has been hindered by the excessive reaction of opinion against the exclusive doctrines of the Humoralists of a past age. Liebig, Prout, Bright and others, have established a firm foundation of well-ascertained facts, upon which it is hoped the superstructure of a new and rational system of humoral pathology will arise, fact by fact, cemented fast by experiment and logical deduction.

